# DNA Damage Mediated S and G_2_ Checkpoints in Human Embryonal Carcinoma Cells

**DOI:** 10.1634/stemcells.2008-0690

**Published:** 2009-03

**Authors:** XiaoQi Wang, Vincent CH Lui, Ronnie TP Poon, Ping Lu, Randy YC Poon

**Affiliations:** a Department of Surgery, The University of Hong KongPokfulam, Hong Kong; b Department of Biochemistry, The Hong Kong University of Science & TechnologyClear Water Bay, Hong Kong

**Keywords:** Embryonal carcinoma cell, Cell cycle, Checkpoint, Ionizing radiation

## Abstract

For mouse embryonic stem (ES) cells, the importance of the S and G_2_ cell cycle checkpoints for genomic integrity is increased by the absence of the G_1_ checkpoint. We have investigated ionizing radiation (IR)-mediated cell cycle checkpoints in undifferentiated and retinoic acid-differentiated human embryonal carcinoma (EC) cells. Like mouse ES cells, human EC cells did not undergo G_1_ arrest after IR but displayed a prominent S-phase delay followed by a G_2_-phase delay. In contrast, although differentiated EC cells also failed to arrest at G_1_-phase after IR, they quickly exited S-phase and arrested in G_2_-phase. In differentiated EC cells, the G_2_-M-phase cyclin B1/CDC2 complex was upregulated after IR, but the G_1_-S-phase cyclin E and the cyclin E/CDK2 complex were expressed at constitutively low levels, which could be an important factor distinguishing DNA damage responses between undifferentiated and differentiated EC cells. S-phase arrest and expression of p21 could be inhibited by 7-hydroxystaurosporine, suggesting that the ataxia-telangiectasia and Rad-3-related-checkpoint kinase 1 (ATR-CHK1), and p21 pathways might play a role in the IR-mediated S-phase checkpoint in EC cells. IR-mediated phosphorylation of ataxia-telangiectasia mutated, (CHK1), and checkpoint kinase 2 were distinctly higher in undifferentiated EC cells compared with differentiated EC cells. Combined with the prominent S and G_2_ checkpoints and a more efficient DNA damage repair system, these mechanisms operate together in the maintenance of genome stability for EC cells. Stem Cells *2009;27:568–576*

## INTRODUCTION

Embryonic stem (ES) cells are defined as having the capacity for both self-renewal and differentiation [1,2]. Such a unique capacity enables ES cell to proliferate extensively while maintaining the potential to differentiate into a wide variety of cell types. Compared with somatic cells, ES cells have many unusual proliferative properties [3], one of which is a distinct cell cycle distribution with a large proportion of cells (approximately 60%) in S-phase and 20% of cells in G_1_- and G_2_-phase in mouse and primate ES cells [4–7]. The cell cycle timing of ES cells also differs from that of somatic cells: the average cell cycle time of a 6.25-dpc epiblast is 9.1 hours and that of a mouse ES cell is 11 hours, compared with NIH3T3 cells (22.1 hours), and mouse embryonic fibroblasts (25.3 hours) [4]. Human ES cells (line H1) also have a shortened G_1_ (2.5-3 hours) and 65% of growing cells are in S-phase, with duration of S-, G_2_-, and M-phase being 8, 4, and 1 hours, respectively [8]. As ES cells differentiate, their cell cycle changes significantly to incorporate a longer G_1_-phase. Thus, ES cells seem to have an extremely rapid cell division, which is due in part to an atypical cell cycle that lacks gap phases and consists mainly of S-phase and M-phase [4]. In murine ES (mES) cells, pRb and p107 are held in a hyperphosphorylated state and do not associate with the E2F family of transcription factors [4,9]. Therefore, the lack of inhibition of E2F by pRb may account for the short G_1_ period of ES cells. When ES cells differentiate, pRb control on G_1_ is imposed, which coincides with the establishment of cell cycle regulated cyclin-dependent kinase (CDK) activities [4,3]. Human ES cells show a decreased dependence on E2F/pRb pathway in regulation of G_1_- to S-phase transition [10], and they express all G_1_-related cyclins (D1, D2, D3, and E) and CDKs (CDK2, CDK4, and CDK6) [11]. In particular, high mRNA level of cyclin D2/CDK4 may contribute to rapid G_1_ progression of human ES cells [8].

Somatic cells respond to DNA damage at different phases of the cell cycle by activating sets of checkpoint pathways. As cell cycle arrest in G_1_ prevents cells with damaged DNA from entering S-phase [12], the G_1_ DNA damage checkpoint is an important mechanism for maintaining genome stability in somatic cells. However, pluripotent cells in mice and primates do not undergo G_1_ growth arrest after DNA damage [6,7,13]. A short G_1_-phase and the absence of a DNA damage-mediated G_1_ checkpoint are thus unique cell cycle characteristics of ES cells. Such features are associated with distinct cell cycle regulation in ES cells: (a) an inactive pRb-E2F pathway [3,4,9]; (b) low expression of cyclin D1 [3,4]; and (c) absence of stress-induced transactivation of p21 [14].

In somatic cells, the S-phase checkpoint is crucial because S-phase is a last line of defense before DNA lesions are converted into heritable mutations: each lesion will inevitably be encountered by the replication machinery and stall DNA replication forks [12]. The G_2_ checkpoint provides extra time to repair and remove DNA lesions before they are passed on to daughter cells [12]. In general, activation of the ataxia-telangiectasia mutated/ataxia-telangiectasia and Rad-3-related-checkpoint kinase 1/checkpoint kinase 2-cell division cycle 25 homolog A (ATM/ATR-CHK1/CHK2-CDC25A) pathway mediates inactivation of the S-phase cyclin E/CDK2 complexes and S-phase arrest [15], and the ATM/ATR-CHK1/CHK2-CDC25C pathway mediates G_2_-phase arrest by maintaining the inactive form of the cyclin B1/CDK1 complex [16,17]. In ES cells, the absence of a G_1_ cell cycle checkpoint has been relatively well-studied. It has also been found that decatenation checkpoint is defective in mES cells and human CD34^+^ progenitor cells [18], resulting in chromosome missegregation in these cells. Thus, the lack of a G_1_ checkpoint and ineffectiveness of decatenation checkpoint increase the importance of S and G_2_ DNA damage checkpoints for genomic integrity in stem cells, which so far are not well-understood. Jirmanova et al. showed that p38α kinase played a critical role for normal S-phase function in mouse ES cells when the ATR/CHK1 pathway is inhibited [19]. However, this study was limited to the caffeine-induced S-phase checkpoint [19]. Recently, p21 has been found to rapidly increase in response to IR in human ES cells, which may further alter E2F/pocked proteins and Histone H4 gene expression. However, it is limited at mRNA level, and how the altered gene expressions affect cell cycle progression is still unclear [20].

ES cells must maintain sufficient genomic integrity to pass an intact genome to the next generation [21]. The DNA repair pathways are involved in successful mammalian gametogenesis and embryo development, with many important DNA repair genes expressed in the early stages of mammalian development [22]. Deficiency in DNA repair genes is associated with increased rates of birth defects, cancer, and reduced lifespan [23]. Recent evidence indicates that deficiency in DNA repair leads to hematopoietic stem cell ageing with loss of reconstitution and proliferation potential [24]. Another protective mechanism of ES cells is their lack of a G_1_ checkpoint following DNA damage. Instead of G_1_ cell cycle arrest, ES cells are prone to undergo apoptosis (increases in apoptosis of 40%) to prevent cells with damaged genomes from contributing to the developing organism [6,14,25]. Therefore, multiple mechanisms including DNA damage detection, DNA repair, cell cycle arrest, and apoptosis operate together to protect genome stability during embryo development [23].

Previous studies of the cell cycle and checkpoints of stem cells have largely been carried out in vitro using ES cell lines. In the presence of leukemia inhibitory factor (LIF), mES cells can be maintained in culture indefinitely as a stem cell population that resemble the pluripotent cell population of the inner cell mass (ICM); thus, mES cells cycle with kinetics comparable with their counterpart in the embryonic epiblast [4,9]. Although human ES cells are not LIF-dependent in culture, it is believed that the basic cell cycle control mechanisms might be conserved between mouse and human ES cells [9]. The NCCIT cell line used in this study is a pluripotent embryonal carcinoma (EC) cell line, which is capable of somatic and extraembryonic differentiation [26]. In vitro, these cells can differentiate in response to retinoic acid (RA) [27]. Although EC and ES cells are derived from different sources of embryo, in culture system, EC cells have some similarities to ES cells in terms of karyotypic changes, adaptation to culture, and teratotumor formation [28]. EC cells are pluripotent, but they might proliferate in a hierarchical stem cell pattern, which could be a limitation for the study of stem cell cycle structure [29].

Rapid proliferation with a high proportion of cells in S-phase is one of the distinct features of stem cells; therefore, the presence of DNA damage-mediated S and G_2_ checkpoints and the regulation of these checkpoints in stem cells are not well understood. In this study, we compared DNA damage mediated S and G2 checkpoints in undifferentiated and differentiated human EC cells.

## MATERIALS AND METHODS

### Cell Culture and Treatments

Human pluripotent EC cells (line NCCIT from ATCC, Manassas, VA, http://www.atcc.org) were cultured in RPM/1640 medium (Invitrogen, Gibco, Carlsbad, CA, http://www.invitrogen.com) containing 10% FBS (Invitrogen) according to ATCC instructions. All experiments were performed in parallel in undifferentiated EC and differentiated EC counterparts. For somatic differentiation of EC cells, 10 μM RA (Sigma, St. Louis, MO, http://www.sigmaaldrich.com) was added to cultures for 7 days with addition of fresh 10 μM of RA at each medium change. Ionizing radiation (IR) was delivered by exposing cells to Cs^137^ in a Gammacell 3,000 (MDS Nordion, Germany). The checkpoint inhibitor 7-hydroxystaurosporine (UCN-01) was kindly provided by Developmental Therapeutics Program, National Cancer Institute (NCI, Bethesda, MD).

### Flow Cytometric Cell Cycle Analysis

Cells were seeded at a density of 4-5 × 10^5^ at least 24 h before experiments. Cells were mock-irradiated or γ-irradiated with 4, 6, or 15 Gy and incubated for the indicated time. The DNA content of propidium iodide (Sigma) stained cell nuclei was determined using a FACScan (Becton Dickinson, San Jose, CA, http://www.bd.com) and data acquisition was performed with CellQuest (Becton Dickinson) software. The percentages of cells in G_1_-, S-, and G_2_/M-phase were calculated using ModFit software (Verity Software House, Topsham, ME). For 5-bromo-2′-deoxyuridine (BrdU) incorporation analysis, cells were pulsed with 10 μM BrdU (Sigma) for 30 minutes before harvesting and fixed in 80% cold ethanol. BrdU content was determined by a FITC-conjugated anti-BrdU antibody (Becton Dickinson) according to the manufacturer's instructions. Propidium iodide-RNase (Sigma) counterstaining was used to determine DNA content.

### Antibodies and Western Blots

Antibodies against cyclin A, E, B1, CDC25A, CDK2, CDC2, p21, p27, and p53 were obtained from Santa Cruz Biotechnology Inc. (Santa Cruz, CA, http://www.scbt.com). Antibodies against phospho-ATM (Ser1981), phospho-CHK1 (Ser345), phospho-CHK2 (Thr68), phospho-CDC25C (Ser216), phospho-p53 (Ser15), phospho-CDC2 (Tyr15), and cleaved caspase three were from Cell Signaling Technology (Beverly, MA, http://www.cellsignal.com). OCT4 antibody was purchased from Chemicon, Millipore (Billerica, MA, http://www.millipore.com). Ten to 30 μg of total protein lysates were loaded and separated on 10% or 12% acrylamide gels and transferred to PVDF membranes. Membranes were incubated with primary antibody overnight at 4°C, HRP-conjugated secondary antibody for 1 hour at room temperature, and protein expression was revealed with ECL reagents (Amersham, GE healthcare UK Limited, U.K., http://www.gehealthcare.com).

### Immunoprecipitation

Total cell lysates (200 μg) were incubated with 2 μl of primary antibody for immunoprecipitation on ice for 1-2 hours, then incubated with 30 μl protein G sepharose (Amersham Biosciences, Sweden, http://www.gehealthcare.com) for 1 hour followed by three washes with lysis buffer. The immune complexes were collected and dissolved in SDS sample buffer for Western blots.

### Immunofluorescence

Undifferentiated and RA-induced differentiated NCCIT cells were seeded and grown for more than 24 hours followed by exposure to 3 Gy of IR. At 0, 1.5, and 30 hours, cells were cyto-spun onto gelatinized slides. Cells were fixed in 4% paraformaldehyde, permeabilized with 0.25% Triton X-100 in PBS, and incubated with antibody (phospho-H2AX Ser139, Cell Signaling Technology, Beverly, MA, http://www.cellsignal.com) at 4°C overnight. Cells were then incubated with FITC-conjugated secondary antibody (Sigma) for 1 hour. Images were captured with a Nikon microscope system. The numbers of γH2AX foci per cell of 1,000 cells from two independent experiments were scored.

### Colony Forming and Cell Viability Assays

Undifferentiated and RA-differentiated EC cells were seeded at a density of 5,000-7,000 cells per 5-cm culture dish. After 40 hours, the cells were exposed to 0, 0.5, 1, or 1.5 Gy of IR. After incubating for another 7 days, colonies were stained by velvet blue and quantified. For cell viability assays, cells were seeded at 1 × 10^4^ per well in 96-well plates and incubated with 2-(2-methoxy-4-nitrophenyl)-3-(4-nitro-phenyl)-5-(2,4-disulfophenyl)-2H-tetrazolium (Cell Counting Kit-8, Dojindo Lab, Japan, http://www.dojindo.com) following manufacturer instructions. The formazan dye generated by the activity of dehydrogenases was measured at 450 nm absorbance and was proportional to the number of living cells.

### TUNEL Assays

For TUNEL analysis, a fluorescence-conjugated in situ cell death detection kit (Roche Diagnostics, Mannheim, Germany, https://www.roche-applied-science.com) was used following the manufacturer's instructions. A total of 1,000-1,500 cells were scored to calculate the percentage of apoptotic cells.

## RESULTS

### Different IR-Mediated Cell Cycle Checkpoints in Undifferentiated and Differentiated EC Cells

**Figure 1:**
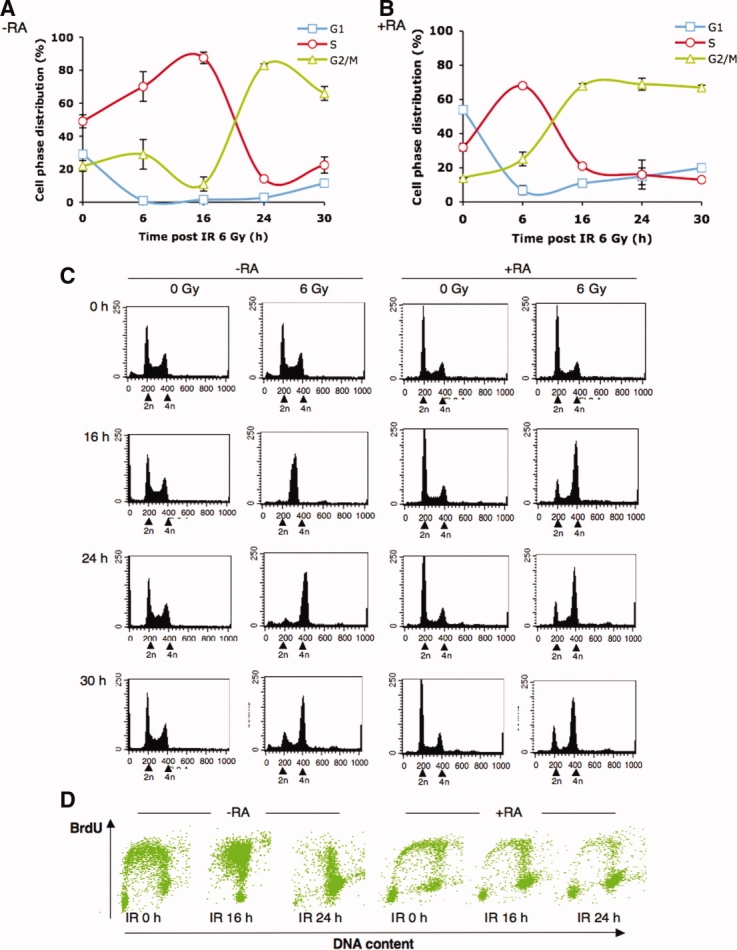
Undifferentiated EC cells underwent prominent S-phase delay followed by G_2_ arrest in response to IR. Undifferentiated and differentiated NCCIT cells were irradiated (6 Gy) followed by flow cytometry analysis of DNA content. Percentages of **(A)** undifferentiated (−RA) and **(B)** differentiated (+RA) NCCIT cells in G_1_, S, and G_2_/M cell cycle phase as a function of time after irradiation (means ± SD; from three independent experiments) were determined using Modfit software. **(C):** Representative histogram of cell cycle distribution in irradiated undifferentiated (−RA) and differentiated (+RA) NCCIT cells at indicated time points. Peaks corresponding to cells with 2*n* and 4*n* DNA content were indicated by arrows. **(D):** Dot plot representation of PI/BrdU double parameter analysis of irradiated undifferentiated (−RA) and differentiated (+RA) NCCIT cells at indicated time points. *x*-axis indicates DNA content and *y*-axis indicates BrdU positive labeling. Abbreviations: BrdU, bromodeoxyuridine; IR, ionizing radiation; RA, retinoic acid.

The absence of a DNA damage-mediated G_1_ cell cycle checkpoint has been observed in mouse ES cells [6,13], bringing up the question of what machinery ES cells employ for to protect genome stability. Using human EC NCCIT cells as a model, we investigated how human EC cells respond to IR-mediated DNA damage. In parallel, DNA damage checkpoint functions were compared with differentiated EC cells. In an asynchronous population, human EC cells displayed a cell cycle phase distribution (Fig. 1A, 1C) that was similar to mouse ES cells [9]. Following 6 Gy of IR, the undifferentiated EC cells did not arrest in G_1_-phase. Instead, the cells displayed a prominent S-phase delay (87.5% ± 3.5%) at 16 hours post-IR, followed by a G_2_-phase delay (83% ± 0.79%, 66% ± 4.2%) at 24 and 30 hours post-IR (Fig. 1A, 1C). Using BrdU labeling, we found that EC cells exhibited a high basal line of S-phase; this fraction was further increased in response to IR, before gradually moving into G_2_-/M-phase (Fig. 1D).

In contrast, RA-induced differentiated EC cells quickly exited S-phase and arrested in G_2_ after DNA damage. The S-phase fraction was 32.5% ± 2.2% at 0 hour, 21% ± 1.3% at 16 hours, 16% ± 8.5% at 24 hours, and 13.5% ± 0.7%, whereas the G_2_ fraction increased from 13.8% ± 0.2% at 0 hour to 68% ± 1.2% at 16 hours, 69% ± 3.5% at 24 hours, and 67% ± 1.4% at 30 hours (Fig. 1B, 1C, 1D). Thus, in response to IR, both undifferentiated and differentiated EC cells displayed a G_2_ arrest response, but undifferentiated EC cells displayed an S delay before they arrested at G_2_-phase.

There was a complete lack of IR-mediated G_1_ cell cycle delay from 6 to 30 hours after IR in human EC cells (Fig. 1A, 1C). BrdU labeling further verified the absence of a G_1_ population in undifferentiated EC cells after IR (Fig. 1D). These results suggest that the lack of a functional G_1_ damage checkpoint is a common phenomenon in pluripotent cells from mice [6,13], primates [7], and humans. Moreover, although the G_1_ population increased when the EC cells differentiated (54% ± 2% in differentiated EC cells vs. 29% ± 0.8% in undifferentiated EC cells; Fig. 1A, 1B), there was no obvious G_1_ growth arrest after IR DNA damage (Fig. 1B, 1C, 1D), indicating that RA-induced differentiation could not establish the G_1_ checkpoint in EC cells.

### Distinct Regulation of Cyclin E/CDK2 After DNA Damage in Undifferentiated and Differentiated EC Cells

**Figure 2:**
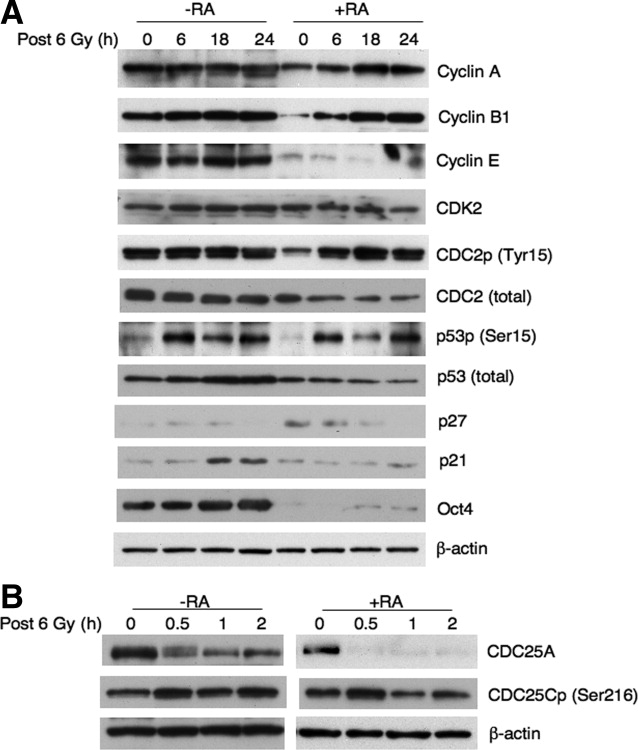
Distinct expression of cell cycle-related proteins in undifferentiated and differentiated embryonal carcinoma cells following ionizing radiation. **(A)** Western blot analysis of cyclins A, E, and B1, CDK2, CDC2, phospho-CDC2 (Tyr15), phospho-p53 (Ser15), p21, p27, OCT4, and **(B)** CDC25A and phospho-CDC25C (Ser216) at various time points after irradiation in undifferentiated (−RA) and differentiated (+RA) NCCIT cells. Abbreviations: CDC25A, cell division cycle 25 homolog A; CDK2, cyclin-dependent kinase 2; RA, retinoic acid.

The above data indicate that although human EC cells lacked the G_1_ DNA damage checkpoint, they seemed to have intact S and G_2_ checkpoints. When EC cells became differentiated, the ability of the cells to undergo IR-mediated S-phase delay was attenuated and G_2_-phase arrest became prominent. We then asked whether there is any difference in the cell cycle machinery between undifferentiated and differentiated human EC cells, which might differentially regulate cell cycle checkpoints. First, there were large differences in the absolute levels of cyclins between undifferentiated and differentiated EC cells. Undifferentiated EC cells expressed high levels of cyclin A, cyclin E, and cyclin B1. The levels of these cyclins decreased dramatically after differentiation (Fig. 2A, at zero timepoint). This phenomenon is consistent with observations in mouse and primate ES cells [3,7,9]. OCT4 level was dramatically reduced or almost depleted in RA-induced EC cells (Fig. 2A), suggesting that the cells were well differentiated.

We next investigated the expression of cell cycle regulators in human EC cells after IR-mediated DNA damage. In undifferentiated EC cells, basal levels of cyclin A, E, B1, and Tyr15-phosphorylated CDC2 were high; they were only slightly enhanced after IR damage and did not show apparent periodicity in accordance with cell cycle progress (Figs. 1A, 2A). Examination of other DNA damage checkpoint regulators, such as phospho-p53 (Ser15), and the CDK inhibitors p21 and p27, the phosphorylation of p53 on ser15 was normal in both undifferentiated and differentiated EC cells. p21 level was low but detectable in both undifferentiated and differentiated cells (Fig. 2A), unlike previous reports in mouse ES cells that p21 levels are constitutively low [19,30]. In response to IR, p21 levels clearly increased in undifferentiated cells but not in differentiated cells. If p21 indeed has a role in cell cycle arrest, it might involve in regulation of the S-phase checkpoint but not induction of a G_1_ checkpoint. Moreover, although IR-mediated CDC25A degradation was very minor in undifferentiated EC cells, it became complete in differentiated EC cells (Fig. 2B). Phospho-CDC25C (Ser216) was enhanced after IR in both undifferentiated and differentiated EC cells (Fig. 2B).

**Figure 3:**
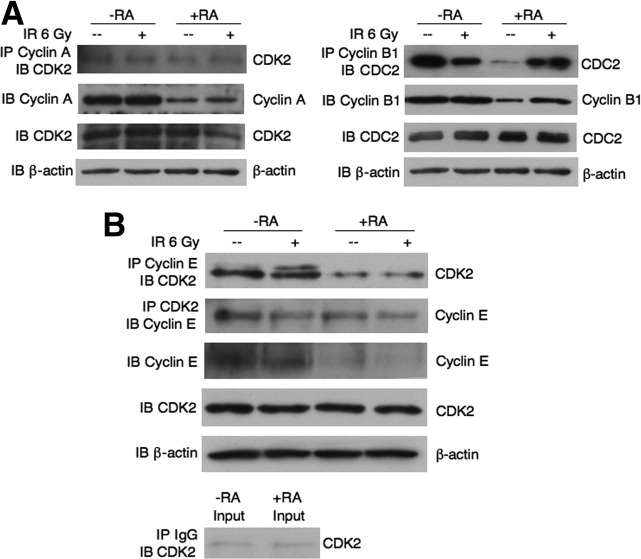
Constitutively low levels of cyclin E and cyclin E/CDK2 complex before and after IR in differentiated NCCIT cells. Cells were untreated or treated with 6 Gy of IR. **(A):** Cyclin A/CDK2 and cyclin B1/CDC2 complexes were analyzed by IP for cyclin A then IB for CDK2; and IP for cyclin B1 then IB for CDC2, respectively. **(B):** Cyclin E/CDK2 complexes were analyzed by IP cylin E then IB CDK2, or IP CDK2 then IB cyclin E. Normal rabbit IgG was used for control immunoprecipitations. Abbreviations: CDK2, cyclin-dependent kinase 2; IB, immunobloting; IP, immunoprecipitating; IR, ionizing radiation; RA, retinoic acid.

In contrast, levels of all the cyclins tested were relatively low in differentiated EC cells before DNA damage. After IR, cyclin B1 was significantly elevated with a parallel increase in Tyr15-phosphorylated CDC2 (Fig. 2A) and cyclin B1/CDC2 complex (Fig. 3A). This coincided with a time after IR of 16-24 hours, when most cells were arrested in G_2_ (Fig. 1B, 1C, 1D). Likewise, cyclin A level was elevated after DNA damage (Figs. 2A, 3A). As cyclin E-CDK2 complexes are key regulators of the G_1_-S transition, we measured cyclin E-associated CDK2 by coimmunoprecipitation. As shown in Figures 2A and 3B, cyclin E levels were constitutively low and cyclin E bound to less CDK2 in differentiated EC cells in comparison with undifferentiated EC cells, particularly absence of responding enhancement in response to IR indicative of potential distinct regulation of cyclin E/CDK2 after DNA damage.

### Robust Activation of Checkpoint Proteins in Undifferentiated EC Cells

**Figure 4:**
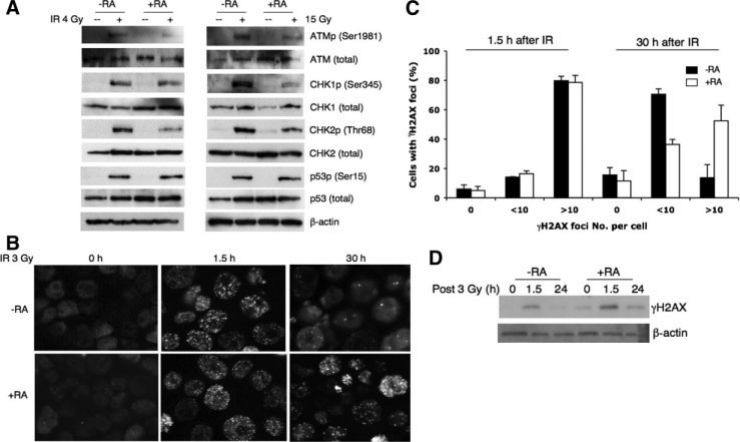
DNA repair is more efficient in undifferentiated embryonal carcinoma cells. **(A):** Undifferentiated (−RA) and differentiated (+RA) NCCIT cells were irradiated with 4 or 15 Gy IR. The total and phosphorylated ATM, CHK1, CHK2, and p53 were measured by Western blot. **(B):** IR-induced DNA damage foci in undifferentiated (−RA) and differentiated (+RA) NCCIT cells at 0, 1.5, and 30 hours were localized by immunofluorescence using anti-γH2AX antibody. **(C):** Quantification of the percentage of cells with 0, <10, and >10 γH2AX foci per nucleus at 0, 1.5, and 30 hours after IR (3 Gy) in undifferentiated (−RA) and differentiated (+RA) NCCIT cells. Data represent means ± SD from two independent experiments. **(D):** Western blot analysis of γH2AX level at 0, 1.5, and 24 hours after IR (3Gy) in undifferentiated (−RA) and differentiated (+RA) NCCIT cells. Abbreviations: ATM, ataxia-telangiectasia mutated; ATMp, phospho-ATM; CHK1, checkpoint kinase 1; CHK1p, phospho-CHK1; CHK2, checkpoint kinase 2; CHK2p, phospho-CHK2; IR, ionizing radiation; p53p, phospho-p53; RA, retinoic acid.

We further compared the activation of components of checkpoint pathways between undifferentiated and differentiated EC cells. Irradiation with 4 Gy or 15 Gy IR rapidly induced the phosphorylation of ATM, CHK1, CHK2, and p53 in both undifferentiated and differentiated EC cells (Fig. 4A). Although the levels of p53 Ser15 phosphorylation were similar in both types of cells, phosphorylation of ATM, and especially activation of CHK1 and CHK2, was distinctly higher in undifferentiated EC cells than in differentiated EC cells (Fig. 4A). These results suggest that undifferentiated EC cells might be primed to respond to DNA damage. Preferential checkpoint activation in EC cells indicated a strong protective mechanism in these cells.

### DNA Damage Repair is More Efficient in Undifferentiated EC Cells

Following double-strand breaks, phosphorylated histone H2AX (also called γH2AX) forms foci at sites of DNA damage, which serve as platforms to recruit DNA repair proteins and cell cycle checkpoint proteins such as ATM and CHK2 [31,32]. We thus determined whether higher checkpoint activity in EC cells leads to more efficient DNA damage repair by monitoring γH2AX foci formation after IR treatment. Undifferentiated and differentiated EC cells were equally susceptible to IR, with more than 90% of cells forming γH2AX foci 1.5 hours after IR. Most cells displayed 10 or more γH2AX foci per nucleus (Fig. 4B, 4C). However, DNA damage foci number was dramatically reduced in undifferentiated EC cells 30 hours after IR. In contrast, the number of γH2AX foci remained high in differentiated EC cells, with 52.45% ± 10.6% of cells displaying >10 γH2AX foci (Fig. 4C). The relatively higher level of γH2AX remaining in differentiated EC cells was further confirmed by immunoblotting (Fig. 4D). Accordingly, the proportion of cells with <10 γH2AX foci was significantly increased in undifferentiated EC cells at 30 hours after IR, indicating that undifferentiated EC cells repaired DNA damage more efficiently than differentiated EC cells (Fig. 4B, 4C). We noticed that DNA damage foci did not completely disappear at 30 hours after IR, suggesting that longer periods may be required for complete repair. Taken together, these data show that the greater activation of ATM, CHK1, and CHK2 in undifferentiated EC cells might result in more efficient DNA damage repair.

### Preferential Checkpoint Activation and DNA Damage Repair as a Protective Mechanism in Undifferentiated EC Cells

**Figure 5:**
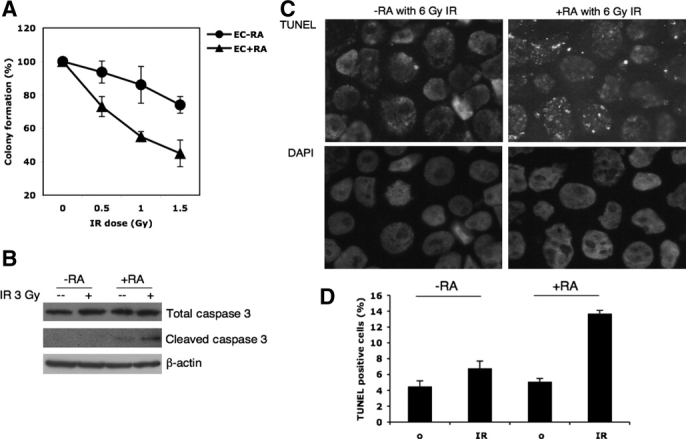
Undifferentiated EC cells showed better cellular survival after IR than differentiated EC cells. **(A):** Undifferentiated (−RA) and differentiated (+RA) NCCIT cells were irradiated with 0, 0.5, 1, and 1.5 Gy of IR and cultured for 7 days. Percentages of NCCIT cells capable of colony formation after irradiation were determined by dividing the number of colonies formed in irradiated cultures by the number of colonies formed in nonirradiated cultures. Values represent means ± SD from two independent experiments and each experiment was performed in triplicate for each dosage of IR treatment. **(B):** Total and cleaved caspase three in irradiated and nonirradiated undifferentiated (−RA) and differentiated (+RA) NCCIT cells (18 hours after IR) were detected by western blot. **(C):** Apoptotic cells in irradiated and nonirradiated undifferentiated (−RA) and differentiated (+RA) NCCIT cells (18 hour after 6 Gy of IR) were detected by TUNEL assay. **(D):** Percentages of apoptotic cells were determined by scoring the numbers of TUNEL positive cells in 1,500 cells from two experiments. Abbreviations: DAPI, 4′-6-diamidino-2-phenylindole; EC, embryonal carcinoma; IR, ionizing radiation; RA, retinoic acid; TUNEL, terminal deoxynucleotidyl transferase-mediated dUTP nick-end labeling.

The ability to repair damaged DNA is critical for cell survival, because unrepaired DNA strand breaks can trigger apoptosis or senescence. We next determined the contributions of preferential activation of DNA damage checkpoints and DNA repair capability to cell survival in EC cells. After exposure to low doses of IR, the survival rates of undifferentiated EC cells by colony formation were 93.8%, 86%, and 78% at treatments of 0.5 Gy, 1 Gy, and 1.5 Gy, respectively, whereas the survival rates of differentiated EC cells were 73%, 55%, and 45% for the same exposures (Fig. 5A), indicating that undifferentiated EC cells had relatively higher survival than differentiated EC cells after IR treatment.

To examine apoptosis in EC cells, the expression of activated caspase-3 and the number of apoptotic cells were examined. Undifferentiated EC cells expressed less activated caspase-3 than differentiated cells, both before and after IR (Fig. 5B). TUNEL assays revealed that undifferentiated EC cells underwent less apoptosis than differentiated cells after IR (Fig. 5C). Thus, preferential checkpoint activation and DNA repair efficiency in undifferentiated EC cells might serve as protective mechanisms in response to DNA damage.

### Biological Importance of the S-Phase Checkpoint

**Figure 6:**
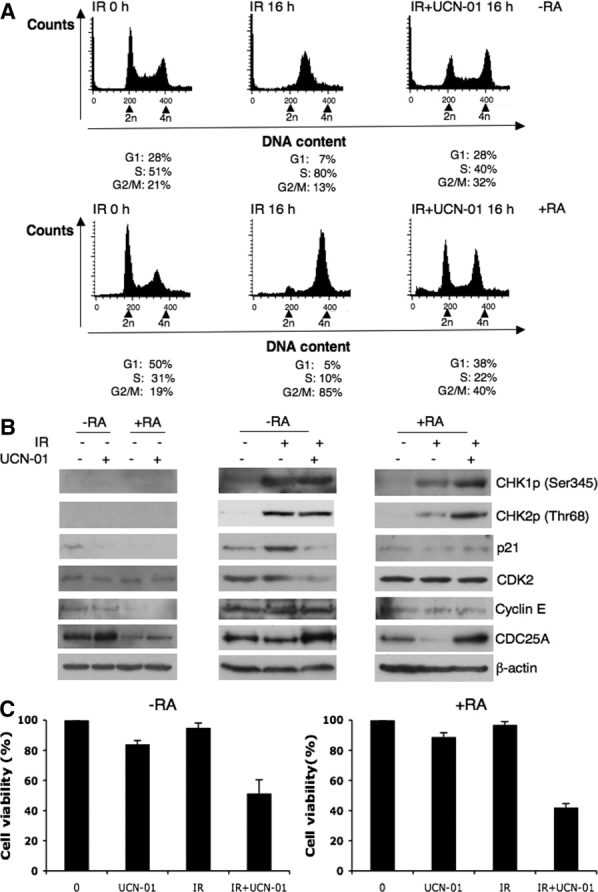
IR-mediated S-phase arrest in undifferentiated embryonal carcinoma cells could be inhibited by UCN-01. Undifferentiated (−RA) and differentiated (+RA) NCCIT cells were treated with 6 Gy of IR. UCN-01 (200 nM) was added to the cultures 15 minutes before irradiation. Cells were harvested and measured for **(A)** cell cycle distributions 16 hours after IR; **(B)** cell cycle and checkpoint related proteins 2 hours after IR and UCN-01 alone by Western blot; **(C)** cell viability 18 hours after IR by MTT (means ± SD from two independent experiments). Abbreviations: CHK1p, phospho-CHK1; CHK2p, phospho-CHK2; CDC25A, cell division cycle 25 homolog A; IR, ionizing radiation; RA, retinoic acid; UCN-01, 7-hydroxystaurosporine.

Cells possess protective mechanisms including DNA damage detection, DNA repair, cell cycle arrest, and apoptosis that operate together to maintain genome integrity. Recent studies suggest that the same pathways regulating the response to DNA damage also operate during normal S-phase [23]. Many types of ES cells, including human EC cells, proliferate actively with a large fraction of cells in S-phase. The S-phase population further increased in response to DNA damage in our experiments. We examined the functional importance of the S-phase checkpoint by S-phase checkpoint abrogation in undifferentiated EC cells. UCN-01, a potent ATR-CHK1 pathway inhibitor, inhibits G_2_ and S-phase checkpoints in cancer cell models [33,34]. As shown in Figure 6A, EC cells were sensitive to UCN-01: the S-phase delay mediated by 6 Gy of IR was largely abolished after UCN-01 treatment, indicating that the ATR-CHK1 pathway was involved in S-phase arrest in these cells. Although CDC25A degradation was clearly prevented (Fig. 6B), IR-induced CDC25A degradation was not complete in undifferentiated EC cells compared with differentiated EC cells (Figs. 2B, 6B), suggesting that CDC25A turnover might not be a major intermediate effector of the S-phase checkpoint. Interestingly, S-phase regulators, such as p21 and CDK2, were also significantly inhibited by UCN-01 (Fig. 6B) in undifferentiated but not in differentiated EC cells; thus, besides the traditional ATR-CHK1-CDC25A pathway, p21 could play a role in regulating the S-phase checkpoint in human EC cells, which requires further study. Moreover, abrogation of S-phase arrest in EC cells resulted in reduction of cellular survival (Fig. 6C), further indicating the functional importance of the S-phase checkpoint as a protective mechanism. In differentiated EC cells, G_2_ cell cycle arrest was also sensitive to UCN-01 (Fig. 6A), likely through inhibition of the ATR-CHK1-CDC25A pathway (Fig. 6B). As a biological consequence, the abrogation of G_2_ arrest led to greater cell death than abrogation of S arrest (Fig. 6C). Thus, to stem cells, S-phase arrest seemed to be more effective for cellular protection when cells are subjected to DNA damage.

## DISCUSSION

A unique characteristic of cell cycle control in stem cells is a short G_1_-phase, with the vast majority of cells in S or G_2_/M. This feature has been conserved throughout vertebrate evolution [7]. Our results indicate that this is also a special feature of cell cycle control for human EC stem cells (Fig. 1). Although the mechanisms that allow pluripotent stem cells to cycle rapidly [4] and devote a high proportion of the cell cycle (approximately 60%) to S-phase [4] is still unclear, it could be due to (a) the required ability to divide constitutively and (b) the consequences of avoiding or shorting G_1_ to prevent differentiation [4,35]. These features are further reflected by the failure of stem cells to activate the G_1_/S checkpoint after DNA damage. Another interesting result of the present study is that the G_1_-phase population increased as EC cells differentiated. However, this simple differentiation induced by RA could not establish a complete G_1_ cell cycle checkpoint on DNA damage even though damage-induced ATM (Ser1981), CHK1 (Ser345), CHK2 (Thr68), and p53 (Ser15) phosphorylation appeared to be normal (Fig. 4A). Therefore, at which stage of development and under what circumstances the G_1_ cell cycle checkpoint is necessary for a cell is still unclear. G_1_ (or early G_1_) regulation is thought to be a sensitive window of cell-fate decision-making in response to extracellular signals; therefore, ES cells try to avoid (mES cells) or shorten (hES cells) G_1_ [35]. It is possible that the absence of a G_1_ checkpoint serves a physiological need for ES cells. If that is the case, the S and G_2_ cell cycle checkpoints become crucial in the maintenance of genome integrity for stem cells.

Our results showed that following IR, undifferentiated human EC cells had better survival rates (Fig. 5) and more efficient DNA repair than their differentiated counterparts (Fig. 4). This may in part be due to the enhanced S-phase delay in undifferentiated EC cells (Fig. 1). When cells lack the G_1_ DNA damage checkpoint, S-phase and G_2_ are the only periods in which cells can arrest the cell cycle to respond to DNA damage. Indeed, we found IR-mediated G_2_ arrest in both undifferentiated and differentiated cells. One conspicuous difference is that undifferentiated EC cells displayed a prominent S-phase delay before entering G_2_ arrest. This IR-mediated S-phase arrest was inhibited by the CHK1 inhibitor UCN-01, resulting in reduced cell viability (Fig. 6). These data imply that S-phase arrest may serve as a cellular protective mechanism. Therefore, the fact that the majority of pluripotent EC cells are in S-phase could not only allow for coping with rapid proliferation, but also provide the machinery needed for S-phase delay when cells are subjected to DNA damage. The prominence of S-phase in the cell cycle of stem cells could provide a checkpoint window for genome integrity and cellular survival.

Mouse ES cells have constitutive cyclin E-CDK2 activity that is independent of cell cycle control [4,9]. The biological significance of this phenomenon is not fully understood. Concomitant with hyperphosphorylation and inactivation of RB, high cyclin E-CDK2 activity effectively reduces the length of G_1_-phase. This leads to rapid proliferation, which is postulated to be an important cell cycle regulation for self-renewal in stem cells [35]. In undifferentiated EC cells, cyclin E/A and their associated CDK2 were expressed at high levels and remained high after DNA damage (Figs. 2A, 3). In contrast, cyclin E and its associated CDK2 levels were much lower in differentiated EC cells, and without further responding increase on DNA damage (Figs. 2A, 3B). The different levels of cyclin E and cyclin E/CDK2 complexes could be important factors in distinguishing cell cycle features of undifferentiated from differentiated EC cells.

Our results indicate that the ATR-CHK1-CDC25A pathway is a significant pathway for the G_2_ DNA damage checkpoint, particularly for differentiated EC cells because IR-induced CDC25A degradation was clearly prevented by the CHK1 inhibitor UCN-01 (Fig. 6B). However, IR-mediated S-phase delay in undifferentiated EC cells occurred through the ATR-CHK1 pathway, but not necessarily the ATR-CHK1-CDC25A pathway because IR-induced CDC25A degradation was very minor in these cells (Figs. 2B, 6B). Moreover, we showed that S-phase-controlling proteins such as p21 and CDK2 could be inhibited by UCN-01. The CDK inhibitor p21 is known to regulate CDK2 by associated with cyclin E/CDK2 and cyclin A/CDK2 complexes [19]. Thus, p21 could play roles in regulating the S-phase checkpoint for human EC cells. It would be interesting to explore whether there is any link between ATR-CHK1 and p21 regulation and the details of how p21 regulates G_1_-S-phase cyclins in human EC cells.

ES cells from mouse, rhesus monkey, and human (e.g., the EC cells in the present study) fail to undergo G_1_ cell cycle arrest after DNA damage. It has been reported that two mechanisms are used to maintain genomic stability after DNA damage in the absence of a G_1_ checkpoint in stem cells: (a) apoptosis to eliminate cells with damaged DNA [6,7,25] and (b) induction of a differentiation program to eliminate cells with damaged DNA from the stem cell pool [36]. However, DNA repair genes have been shown to be expressed in the early stages of mammalian development. In humans, expression of ATM, *BRCA1*/2, and *TP53* can be detected as early as the preimplantation embryo [23]. The expression of DNA repair genes can determine whether or not the embryo survives following genotoxic stress [23], which would be a more efficient and applicable mechanism than constantly undergoing apoptosis during the long process of development. Our results demonstrate that human EC cells displayed normal activating phosphorylation of ATM, CHK1, CHK2, and p53 (Fig. 4A). Accordingly, EC cells also underwent S and G_2_ cell cycle arrest in response to IR-induced DNA damage (Fig. 1). More importantly, EC cells displayed efficient DNA damage repair (Fig. 4B, 4C). These mechanisms appear to be more robust in EC cells than in their differentiated counterparts. Therefore, we postulate that the S and G_2_ checkpoints, as well as DNA repair and apoptosis, operate together in the maintenance of genome stability for stem cells.

## CONCLUSION

A short G_1_-phase and the absence of G_1_ DNA damage checkpoint are the special cell cycle features common in mouse ES, rhesus monkey ES, and human EC cells (present study). Human EC cells undergo prominent S-phase delay followed by G_2_ arrest in response to IR. After differentiation, cells mainly display a G_2_ arrest, due to the low expression of cyclin E and cyclin E/CDK2 complex. Thus, given that the absence of a G_1_ checkpoint serves a physiological need for self renewal of ES cells, the S and G_2_ cell cycle checkpoints, in combination with more efficient DNA damage repair, are necessary for the maintenance of genome integrity in stem cells.
